# Molecular cytogenetic identification and nutritional composition evaluation of newly synthesized *Triticum turgidum*-*Triticum boeoticum* amphiploids (AABBA^b^A^b^)

**DOI:** 10.3389/fpls.2023.1285847

**Published:** 2023-12-07

**Authors:** Xin Liu, Xiaomei Jiang, Junqing Zhang, Hong Ye, Mang Shen, Lei Wu, Yongping Miao, Longyu Chen, Ke Zhou, Ming Hao, Bo Jiang, Lin Huang, Shunzong Ning, Xuejiao Chen, Xue Chen, Dengcai Liu, Lianquan Zhang

**Affiliations:** ^1^ State Key Laboratory of Crop Gene Exploration and Utilization in Southwest China, Sichuan Agricultural University, Chengdu, China; ^2^ Triticeae Research Institute, Sichuan Agricultural University, Chengdu, China

**Keywords:** *Triticum boeoticum*, synthetic amphiploid, blue aleurone, anthocyanins, amino acid, micronutrients, PCA

## Abstract

*Triticum boeoticum* Boiss. (A^b^A^b^, 2n = 2x = 14) is a wheat-related species with the blue aleurone trait. In this study, 18 synthetic *Triticum turgidum*-*Triticum boeoticum* amphiploids were identified, which were derived from crosses between *T. boeoticum* and *T. turgidum*. Three probes (Oligo-pTa535, Oligo-pSc119.2, and Oligo-pTa713) for multicolor fluorescence *in situ* hybridization (mc-FISH) were combined with genomic *in situ* hybridization (GISH) to identify chromosomal composition. Seven nutritional indices (anthocyanins, protein, total essential amino acids TEAA, Fe, Zn, Mn and Cu) were measured, and the nutritional components of 18 synthetic amphiploids were comprehensively ranked by principal component analysis (PCA). The results showed that all three synthetic amphiploids used for cytological identification contained 42 chromosomes, including 14 A, 14 B, and 14 A^b^ chromosomes. The average anthocyanin content was 82.830 μg/g to 207.606 μg/g in the whole meal of the 17 blue-grained lines (Syn-ABA^b^-1 to Syn-ABA^b^-17), which was obviously higher than that in the yellow-grained line Syn-ABA^b^-18 (6.346 μg/g). The crude protein content was between 154.406 and 180.517 g/kg, and the EAA content was 40.193-63.558 mg/g. The Fe, Zn, Mn and Cu levels in the 17 blue-grained lines were 60.55 to 97.41 mg/kg, 60.55-97.41 mg/kg, 35.11 to 65.20 mg/kg and 5.74 to 7.22 mg/kg, respectively, which were higher than those in the yellow-grained line. The contribution of the first three principal components reached 84%. The first principal component was mainly anthocyanins, Fe, Zn and Mn. The second principal component contained protein and amino acids, and the third component contained only Cu. The top 5 *Triticum turgidum*-*Triticum boeoticum* amphiploids were Syn-ABA^b^-11, Syn-ABA^b^-17, Syn-ABA^b^-5, Syn-ABA^b^-8 and Syn-ABA^b^-4. These amphidiploids exhibited the potential to serve as candidates for hybridization with common wheat, as indicated by comprehensive score rankings, toward enhancing the nutritional quality of wheat.

## Introduction

Wheat holds significant importance as a primary energy source in many regions globally. Its unique processing qualities facilitate the preparation of various products, such as bread, cookies, pasta, and noodles (John and Babu, 2021). In this era of rapid economic growth, the nutritional perspective on food security is gaining increasing attention (Li et al., 2006; Tian et al., 2018). The development of biofortified colored wheats (black, purple, and blue) provides nutritional and functional health benefits to the already energy-rich wheat (Garg et al., 2022). Currently, the pursuit of high-quality wheat is equally important as that of high-yielding wheat, and biofortified colored wheat offers a novel opportunity.

Anthocyanins are plant secondary metabolites belonging to the polyphenol class of natural water-soluble pigments. They contribute to the vibrant colors observed in various parts of plants, such as the fruits, leaves, seed coat, and flowers, with hues ranging from orange−red to blue−violet (Ficco et al., 2014; Khlestkina et al., 2015; Wallace and Giusti, 2015). Anthocyanins have strong antioxidant potential, as they protect cells from free radical damage by neutralizing and scavenging free radicals (Cerletti et al., 2017). Moreover, they can disrupt the growth cycle of cancer cells. Studies have demonstrated that anthocyanins can also prevent heart disease, inflammation, and cancer and exhibit anti-aging and anti-diabetes properties (Bagchi et al., 2004; Shipp and Abdel-Aal, 2010; Lin et al., 2017).

Micronutrient deficiencies in the human diet have long been a serious concern in global public health (Bhullar and Gruissem, 2013), particularly for grain-based diets, which not only lack a variety of micronutrients but also have micronutrients with reduced bioavailability to the human body. Malnutrition resulting from micronutrient deficiencies, especially zinc and iron deficiencies, affects a significant portion of the global population, with iron deficiency-related anemia being one of the most prevalent health disorders worldwide (Cakmak, 2008; Gupta et al., 2021; Loskutov and Khlestkina, 2021). The World Health Organization (WHO) estimates that over 2 billion people worldwide experience deficiencies in Fe/Zn (https://www.who.int/health-topics/micronutrients#tab=tab_1). Biofortification, which involves increasing the micronutrient content and concentration of cereals, is an important approach to improving nutritional quality. Improving the levels of micronutrients such as iron and zinc in wheat through the use of breeding technology is considered an effective strategy for increasing the intake of essential micronutrients via food (Morgounov et al., 2007).

*T. boeoticum* Boiss. (A^b^ A^b^, 2n=2x=14) is an important source of the blue aleurone trait (Zeven, 1991), with the associated candidate gene being *TbMYC4A* (Liu et al., 2021). *T. boeoticum* Boiss. has attracted the interest of consumers and the food industry due to its beneficial nutritional characteristics. It exhibits low dietary fiber content but is abundant in protein, lipids (mainly unsaturated fatty acids), fructans, carotenoids, and micronutrients (including iron and zinc) (Hidalgo and Brandolini, 2014; Lachman et al., 2017). *T. boeoticum* Boiss. exhibits greater tolerance to diverse environmental stressors than common wheat, rendering it a valuable genetic reservoir for fortifying stress resistance in wheat cultivars (Budak et al., 2013). *Nax2* and *Nax1* represent salt-tolerance genes exclusive to *T. boeoticum* Boiss.; incorporating them into common wheat amplifies its salt tolerance (Munns et al., 2012; Tounsi et al., 2016). To date, three powdery mildew resistance genes—*Pm25, PmTb7A.1*, and potentially allelic variant *PmTb7A.2* have been discerned in *T. boeoticum* Boiss., the latter possibly being linked to *Pm1* (Shi et al., 1998; Elkot et al., 2015). Successfully, resistance genes *Sr22* (for leaf rust) and *QYrtb.pau-5A*, along with *YrZ15-1370* (for stripe rust), have been introduced into common wheat (Paull et al., 1994; Chhuneja et al., 2008; Zhang et al., 2021). Artificial amphiploids play a significant role in wheat breeding programs as well as in genetic and evolutionary research (Gill et al., 1988; Megyeri et al., 2011; Ahmed et al., 2014; Badaeva et al., 2016; Li et al., 2018). To overcome the challenge of obtaining offspring from *T. boeoticum* Boiss and common wheat, we developed 17 new blue-grained synthetic *Triticum turgidum-Triticum boeoticum* amphiploids (AABBA^b^A^b^, 2n = 6x = 42). Cytological identification and nutritional characteristic analysis were conducted on these lines, which are crucial for transferring favorable genes from *T. boeoticum* Boiss. to common wheat, facilitating the improvement of the latter with beneficial traits.

## Materials and methods

### Plant materials

Seventeen new blue-grained synthetic *Triticum turgidum*-*Triticum boeoticum* amphiploids, Syn-ABA^b^-1 to Syn-ABA^b^-17 (AABBA^b^A^b^, 2n = 6x = 42), and one yellow-grained synthetic *Triticum turgidum*-*Triticum boeoticum* amphiploid, Syn-ABA^b^-18 (AABBA^b^A^b^, 2n = 6x = 42), were used in this study (**Table 1**). Eighteen amphiploids and their parents were sown at the Wenjiang Experimental Station of Sichuan Agricultural University in October 2022. Individual plants were grown 10 cm apart within rows, with 30 cm between rows of 1 m long. 182.25 kg of N-P_2_O_5_-K_2_O compound fertilizer with a ratio of 15:15:15 is added per acre. The thousand kernel weight of the 18 amphiploids ranged from 34.77 to 64.30g (**Table 1**), and the grains displayed a relatively plump shape. A suitable quantity of seeds was randomly selected for the determination of nutritional composition.

**Table 1:** Materials used in this study.

Code	Pedigree	Genome	Phenotype	Thousand kernel weight (g)
Syn-ABA^b^-1	AS2291×PI 427514 S_2_	AABBA^b^A^b^	Blue	41.89
Syn-ABA^b^-2	PI352367×PI 427696 S_2_	AABBA^b^A^b^	Blue	48.55
Syn-ABA^b^-3	PI352369×PI 427514 S_2_	AABBA^b^A^b^	Blue	47.13
Syn-ABA^b^-4	PI355490×PI 427696 S_2_	AABBA^b^A^b^	Blue	39.14
Syn-ABA^b^-5	PI184526×PI 427866 S_2_	AABBA^b^A^b^	Blue	51.07
Syn-ABA^b^-6	PI352514×PI 427514 S_2_	AABBA^b^A^b^	Blue	61.71
Syn-ABA^b^-7	AS313×PI 427506 S_2_	AABBA^b^A^b^	Blue	64.30
Syn-ABA^b^-8	AS2308×PI 427506 S_2_	AABBA^b^A^b^	Blue	51.93
Syn-ABA^b^-9	AS2310×PI 427514 S_2_	AABBA^b^A^b^	Blue	47.27
Syn-ABA^b^-10	AS2378×PI 427506 S_2_	AABBA^b^A^b^	Blue	63.80
Syn-ABA^b^-11	AS2381×PI 427506 S_2_	AABBA^b^A^b^	Blue	58.73
Syn-ABA^b^-12	AS2268×PI 427506 S_2_	AABBA^b^A^b^	Blue	53.70
Syn-ABA^b^-13	AS2334×PI 427514 S_2_	AABBA^b^A^b^	Blue	51.50
Syn-ABA^b^-14	Langdon×PI 427864 S_2_	AABBA^b^A^b^	Blue	44.47
Syn-ABA^b^-15	Langdon ×PI 427866 S_2_	AABBA^b^A^b^	Blue	47.47
Syn-ABA^b^-16	Langdo ×*T. boeoticum* (70) S_3_	AABBA^b^A^b^	Blue	48.27
Syn-ABA^b^-17	Langdon ×*T. boeoticum* (5) S_3_	AABBA^b^A^b^	Blue	41.47
Syn-ABA^b^-18	Langdon ×*T. boeoticum* (3) S_3_	AABBA^b^A^b^	Yellow	34.77

AS codes refer to the materials provided by Triticeae Research Institute, Sichuan Agricultural University. PI codes refer to the materials from USDA-ARS, NSGC (https://npgsweb.arsgrin.gov/gringlobal/search.aspx?). *T. boeoticum* (3), *T. boeoticum* (5) and *T. boeoticum* (70) was provided by CIMMYT (International Maize and Wheat Improvement Center).

### Cytological observation

Multicolor fluorescence *in situ* hybridization (mc-FISH) was performed using the methods described by Tang et al. (2014). Oligo-pTa535, Oligo-pSc119.2, and Oligo-pTa713 were utilized as probes to distinguish individual chromosomes of Syn-ABA^b^-1 to Syn-ABA^b^-18. All probes were labeled with either FAM (6-carboxyfluorescein) or TAMRA (6-carboxytetramethylrhodamine) by Tsingke Biotechnology Co., Ltd. Genomic *in situ* hybridization (GISH) was employed to identify the A and B genomes of synthetic amphiploids. The total genomic DNA of *T. boeoticum* G52 (A genome) was labeled with an Atto488 NT labeling kit (green) (Jena Bioscience) as a probe, and the total genomic DNA of *A. speltoides* (B genome) was used for blocking. The identification of A^b^ chromosomes was performed as described by Feng et al. (2020). Hybridization signals were visualized and captured using an Olympus BX-63 epifluorescence microscope equipped with a Photometric SenSys DP70 CCD camera (Olympus, Tokyo, Japan).

### PCR amplification

Genomic DNA was extracted from fresh leaves using a plant genomic DNA kit (Tsingke Biotechnology Co., Ltd.). To identify the *T. boeoticum* blue aleurone layer 2 (*Ba2*) candidate gene (Liu et al., 2021), the *TbMYC4Aa1-F* and *TbMYC4Aa1-R* (forward, 5′- CGCTATCAGCTCCCAGTCAG -3′; reverse, 5′- CATCCTTCCACGCCAGAACT-3′) primers were used. The PCR system and amplification program for primers used in the study were as follows: 1 µl of DNA (200 ng/ml), 0.5 µl of F/R primers (10 mM), 5 µl of 2× *Taq* Master Mix (Tsingke Biotechnology Co., Ltd.), and double-distilled water to 10 µl. The PCR products were examined by 2% agarose electrophoresis.

### Total anthocyanin content

Total anthocyanins in the seeds of the synthetic amphiploids were extracted according to the method described by Wang et al. (2016). All samples were ground in liquid nitrogen and crushed, and approximately 0.5 g of sample powder was accurately weighed for the experiment. To determine the total anthocyanin content (TAC), the absorbance was measured at 530 nm using a microplate reader (Multiskan GO, Thermo Scientific, MA, USA). A standard curve was constructed for cyanidin 3-sophoroside chloride (Sigma) by also measuring the absorbance at 530 nm. The total anthocyanin content of the synthetic amphiploids was quantified in cyanidin 3-sophoroside chloride equivalents.

### Crude protein and amino acid determination

The protein content was analyzed according to the Chinese National Standards: GB 5009.5-2016 (China) by using the Kjeldahl method. Accurately weigh 0.2g of each sample for measurement. A factor of 6.25 was used to calculate the crude protein content (Salo-väänänen and Koivistoinen, 1996).

Quantitative analysis of amino acids was performed using electrospray ionization ultra-performance liquid chromatography tandem mass spectrometry (UPLC−MS/MS) (Waterval et al., 2009). Weigh 0.05g of each sample for quantitative and qualitative analysis of amino acids. Methanol, acetonitrile, and formic acid were purchased from ANPEL. Hydrochloric acid was purchased from Sinoreagent. AccQ•Tag reagent was purchased from Waters.

The sample extracts were analyzed using a UPLC–Orbitrap-MS system (UPLC, Vanquish; MS, QE). The UPLC analytical conditions were as follows: column, Waters ACQUITY UPLC BEH C18 (1.7 μm, 50*2.1 mm); column temperature, 55°C; flow rate, 0.5 mL/min; injection volume, 1 μL; solvent system, water (0.1% formic acid):acetonitrile (0.1% formic acid); gradient program, 95:5 V/V at 0 min, 90:10 V/V at 5.5 min, 75:25 V/V at 7.5 min, 40:60 V/V at 8 min, 95:5 V/V at 8.5 min, 95:5 V/V at 13 min. HRMS data were recorded on a Q Exactive hybrid Q–Orbitrap mass spectrometer equipped with a heated ESI source (Thermo Fisher Scientific) utilizing the full MS acquisition methods. The ESI source parameters were set as follows: spray voltage, 3 kV; sheath gas pressure, 40 arb; aux gas pressure, 10 arb; sweep gas pressure, 0 arb; capillary temperature, 320°C; and aux gas heater temperature, 350°C.

Data were acquired on the Q Exactive instrument using Xcalibur 4.1 (Thermo Scientific) and processed using TraceFinder™4.1 Clinical (Thermo Scientific). The quantified data were output in Excel format. The data are available via ProteomeXchange with identifier PXD046077.

### Amino acid analysis

The amino acid ratio coefficient method evaluates food protein content by calculating the ratio of amino acid(s) (RAA), the amino acid ratio coefficient (RC), and the amino acid ratio coefficient score (SRC) in the sample. The RAA, RC, and SRC were calculated according to the following formula from the FAO/WHO/UNU (2007).


$$\text{RAA}=\frac{Amount\;of\;amino\;acid\;per\;test\;protein}{Amount\;of\;amino\;acid\;per\;test\;protein\;in\;reference\;pattern}$$

$$\text{RC=}\frac{RAA}{RAAaverage}$$

SRC=100-CVx100

where CV is the coefficient of variation of the RC.

### Micronutrients analysis

The whole meal micronutrient concentrations (Fe, Zn, Cu, Mn) were determined using inductively coupled plasma atomic emission spectroscopy (ICP−OES, Thermo Scientific iCAP6300, USA) at wavelengths of 259.5 nm (Fe), 213.8 nm (Zn), 324.7 nm (Cu), and 259.3 nm (Mn), following the guidelines of the National Standard for Food Safety GB 5009.268-2016 (China). A 0.5 g sample was accurately weighed for the experiment.

### Principal component analysis

Initially, the correlations between diverse nutritional indicators within the samples were computed. Following this, principal component analysis (PCA) was performed, and components with eigenvalues surpassing 1 were isolated as principal components. Subsequently, a comprehensive evaluation model was established to compute the composite scores for the 18 synthetic amphiploids. PCA was performed using the R package psych v2.3.6 (Revelle and Revelle, 2015). Images in the analysis process were generated using the R package ggplot2 v.2.2.1 (Wickham, 2011) for plotting. Differences were considered significant at p ≤ 0.05.

### Statistical analysis

All the above nutrients were analyzed in triplicate, and all analyses were carried out in SPSS 20.0 (SPSS 20.0 for Windows; SPSS Inc., Chicago, IL, USA). Differences in nutritional indicators between blue-grained and yellow-grained amphidiploids were evaluated by independent sample t-test. Significant differences were considered at p<0.05.

## Results

### Chromosomal observations

**Figure 1:**
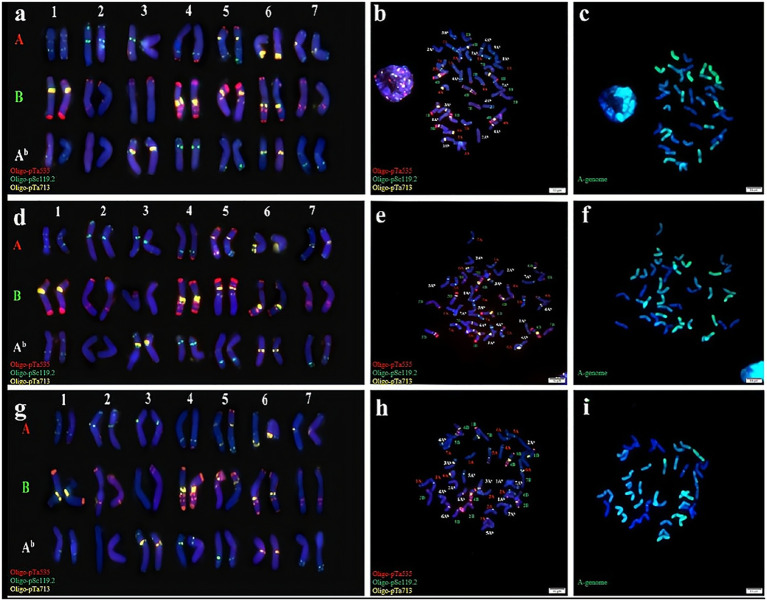
Cytogenetic identification of synthetic amphiploids Syn-ABA^b^-14, Syn-ABA^b^-15 and Syn-ABA^b^-16. FISH identification of **(A, B)**, Syn-ABA^b^-14; **(D, E)**, Syn-ABA^b^-15; and **(G, H)**, Syn-ABA^b^-16 using the three synthetic oligonucleotide probes Oligo-pTa535 (green), Oligo-pSc119.2 (red), and Oligo-pTa713 (yellow). Panels **(C, F, I)** show the GISH patterns of the metaphase chromosomes of Syn-ABA^b^-14, Syn-ABA^b^-15 and Syn-ABA^b^-16. *T. boeoticum* genomic DNA (green) was used as a probe for GISH.

Cytological identification was conducted by analysis of the chromosomes of three synthetic *Triticum turgidum*-*Triticum boeoticum* amphiploids (Syn-ABA^b^-14 to Syn-ABA^b^-16). Subsequently, mc-FISH identification was conducted to clarify their chromosomal composition. To enhance signal acquisition, we used Oligo-pTa535 (green), Oligo-pSc119.2 (red), and Oligo-pTa713 (yellow) as probes for identifying the three lines. Oligo-pSc119.2 effectively identified the chromosomes of the B genome, exhibiting signals on chromosomes 4A and 5A as well. Oligo-pTa535 exhibited distinctive signals at various locations across all A genomes, concurrently identifying chromosomes 1A^b^, 2A^b^, 3A^b^, 4 A^b^, 5 A^b^, and 7 A^b^ within the A^b^ genome; however, the signal intensity was not particularly strong. In contrast, the probe Oligo-pTa713 clearly exhibited noticeable signals on chromosomes 3A^b^ and 6A^b^ within the A^b^ genome. These three probes successfully discriminated the 42 chromosomes of synthetic amphiploids (**Figures 1A, B, D, E, G, H**). The GISH results revealed that these three amphiploids contained 28 A genome and A^b^ genome chromosomes (green) (**Figures 1C, F, I**). In summary, these amphiploids contained 14 A, 14 B, and 14 A^b^ chromosomes.

### Blue aleurone layer 2 candidate gene PCR testing

**Figure 2:**
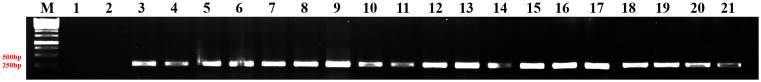
Distribution of *TbMYC4A* in Syn-ABA^b^-1 to Syn-ABA^b^-17. Lanes 1 and 2: *T. turgidum*; lanes 3 and 4: *T. boeoticum*; lanes 5 to 21 correspond to materials from Syn-ABA^b^-1 to Syn-ABA^b^-17, respectively.

The objective was to ascertain the presence of the candidate gene *TbMYC4A* from *T. boeoticum* within the 17 blue-grained amphiploids. For this purpose, specific primers (*TbMYC4Aa1-F* and *TbMYC4Aa1-R*) were employed in the testing process, utilizing *T. turgidum* (negative control) and *T. boeoticum* (positive control) as controls. The results revealed that the 17 blue-grained amphiploids, akin to *T. boeoticum*, exhibited amplification of a 310 bp band (**Figure 2**). In contrast, *T. turgidum* did not display this band, suggesting that the 17 blue-grained amphiploids harbored the candidate gene *TbMYC4A* from *T. boeoticum*.

### Total anthocyanin content

**Figure 3:**
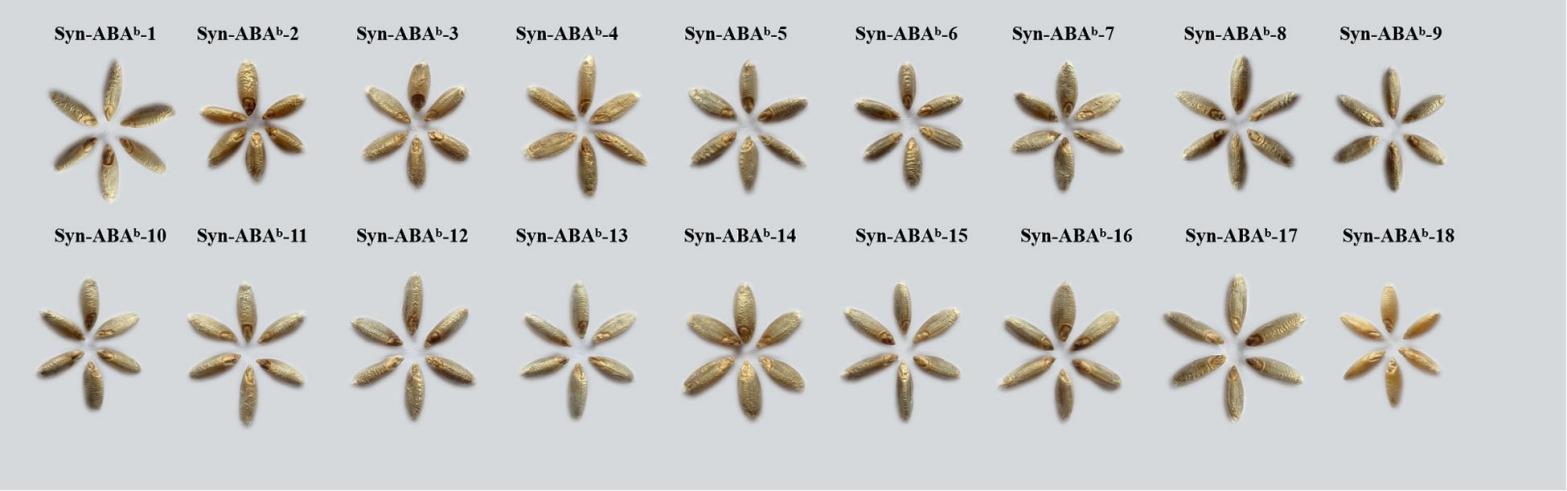
Seed samples of 18 synthetic amphiploids.

The grains of Syn-ABA^b^-1 to Syn-ABA^b^-17 were noticeably blue compared to those of Syn-ABA^b^-18, indicating the inheritance of the blue aleurone trait from *T. boeoticum* (**Figure 3**). After measurement, the TAC of the 17 blue-grained amphiploids ranged from 82.830 μg/g to 207.606 μg/g, which was significantly higher than that of Syn-ABA^b^-18 (yellow-grained) (6.346 μg/g) (**Table 2**).

**Table 2:** The total anthocyanin content of the synthetic hexaploid lines.

Code	TAC (μg/g)
Syn-ABA^b^-1	118.847
Syn-ABA^b^-2	115.813
Syn-ABA^b^-3	179.386
Syn-ABA^b^-4	200.723
Syn-ABA^b^-5	122.411
Syn-ABA^b^-6	124.498
Syn-ABA^b^-7	97.429
Syn-ABA^b^-8	98.903
Syn-ABA^b^-9	115.054
Syn-ABA^b^-10	87.306
Syn-ABA^b^-11	103.156
Syn-ABA^b^-12	131.026
Syn-ABA^b^-13	85.967
Syn-ABA^b^-14	82.831
Syn-ABA^b^-15	139.319
Syn-ABA^b^-16	87.394
Syn-ABA^b^-17	207.600
Syn-ABA^b^-18	6.346

### Crude protein and amino acid analysis

The crude protein content of the 18 amphiploids ranged from 154.406 g/kg to 180.517 g/kg (**Table 3**). Notably, the crude protein content of 12 of these samples surpassed 170 g/kg. Only one line exhibited a protein content lower than that of Syn-ABA^b^-18.

**Table 3:** The crude protein content of the synthetic amphiploids.

Code	Total nitrogen content (g/kg)	Crude protein content (g/kg)
Syn-ABA^b^-1	27.494	171.839
Syn-ABA^b^-2	28.389	177.430
Syn-ABA^b^-3	27.840	174.000
Syn-ABA^b^-4	27.330	170.812
Syn-ABA^b^-5	27.948	174.676
Syn-ABA^b^-6	28.170	176.064
Syn-ABA^b^-7	28.439	177.741
Syn-ABA^b^-8	27.120	169.501
Syn-ABA^b^-9	27.547	172.169
Syn-ABA^b^-10	26.864	167.899
Syn-ABA^b^-11	28.107	175.670
Syn-ABA^b^-12	27.103	169.391
Syn-ABA^b^-13	24.705	154.406
Syn-ABA^b^-14	26.590	166.190
Syn-ABA^b^-15	27.214	170.091
Syn-ABA^b^-16	28.883	180.517
Syn-ABA^b^-17	28.130	175.811
Syn-ABA^b^-18	26.400	165.002

The amino acid content of the 18 amphiploids is presented in **Table S1**. These amphiploids exhibit similar amino acid profiles, encompassing seventeen types of amino acids, of which seven are essential amino acids (EAAs). Among these, the most abundant amino acids closely resemble those found in common wheat, specifically glutamic acid and proline. In comparison with the EAAs outlined in the reference protein pattern recommended by the FAO/WHO/UNU (2007), lysine emerged as the first limiting amino acid in the examined material, with RAA scores ranging from 0.55 to 0.88 across the 18 amphiploids. According to the theory of amino acid balance, the nutritional value of wheat increases as the SRC approaches 100. The SRC values of the 18 amphiploids ranged from 57.60 to 64.57, indicating a relatively well-balanced protein quality (**Table S2**).

### Determination of the micronutrients

**Table 4** presents the microelement content in the 18 amphiploids. Among the four elements measured, the levels in the 17 blue-grained amphiploids were higher than those in the yellow-grained amphiploid. The iron (Fe) content in the blue-grained materials ranged from 60.55 to 97.41 mg/kg, representing a 33.48-114.70% increase compared to the yellow-grained amphiploid. Regarding the zinc (Zn) content, the range was 39.18-57.82 mg/kg, reflecting an increase of 33.48-97.01%. The manganese (Mn) content, ranging from 35.11 to 65.20 mg/kg, exhibited an increase of 42.07-163.87%. Last, the copper (Cu) content ranged from 5.74 to 7.22 mg/kg, indicating an increase of 25.28-57.62%.

**Table 4:** The micronutrient content of the synthetic amphiploids.

Code	Fe(mg/kg)	Zn(mg/kg)	Mn(mg/kg)	Cu(mg/kg)
Syn-ABA^b^-1	67.91 ± 3.51**	51.47 ± 4.05*	53.22 ± 4.31**	6.31 ± 0.23**
Syn-ABA^b^-2	77.58 ± 8.16*	52.34 ± 2.9**	60.4 ± 2.42**	6.78 ± 0.37**
Syn-ABA^b^-3	64.79 ± 6.64*	52.49 ± 2.15**	50.92 ± 3.48**	7.01 ± 0.34**
Syn-ABA^b^-4	73.59 ± 7.04*	48.38 ± 0.66**	51.75 ± 4.88*	6.14 ± 0.29**
Syn-ABA^b^-5	69.08 ± 4.39**	43.76 ± 1.23**	65.2 ± 0.8**	4.9 ± 0.06*
Syn-ABA^b^-6	73.49 ± 6.74*	49.77 ± 7.35*	48.15 ± 4.65**	6.94 ± 0.31**
Syn-ABA^b^-7	81.80 ± 8.5*	51.01 ± 3.26**	46.33 ± 4.68**	7.22 ± 0.23**
Syn-ABA^b^-8	67.49 ± 5.43*	46.71 ± 0.93**	50.41 ± 2.43**	6.47 ± 0.22**
Syn-ABA^b^-9	66.62 ± 9.71*	40.65 ± 1.75**	35.11 ± 2.66*	5.74 ± 0.14**
Syn-ABA^b^-10	60.55 ± 3.7*	39.18 ± 3.5*	44.59 ± 3.85**	6.16 ± 0.5*
Syn-ABA^b^-11	74.91 ± 1.13*	51.75 ± 3.14**	59.46 ± 2.26**	7.16 ± 0.29**
Syn-ABA^b^-12	67.47 ± 7.76*	44.79 ± 2.61**	47.76 ± 1.63**	6.93 ± 0.29**
Syn-ABA^b^-13	66.8 ± 5.81*	41.53 ± 1.08**	46.41 ± 0.37**	6.48 ± 0.14**
Syn-ABA^b^-14	65.76 ± 3.12**	41.98 ± 1.72**	46.78 ± 1.38**	5.9 ± 0.3*
Syn-ABA^b^-15	75.22 ± 6.21*	41.94 ± 2.38*	60.47 ± 4.63**	5.48 ± 0.31*
Syn-ABA^b^-16	87.93 ± 6.45*	50.23 ± 1.31**	54.82 ± 2.2**	6.36 ± 0.17**
Syn-ABA^b^-17	97.41 ± 4.44**	57.82 ± 0.8**	46.46 ± 2.69**	5.54 ± 0.11**
Syn-ABA^b^-18	45.36 ± 5.42	29.35 ± 3.84	24.71 ± 4.47	4.58 ± 0.18

*Means the contents were significantly different (p < 0.05) with Syn-ABA^b^-18, **means (p < 0.01).

### Principal component analysis

The contribution of the first three principal components reached 84% (**Table S3**), representing most of the index information, and could be used as a comprehensive index to evaluate the nutritional quality of the 18 materials. The maximum variance contribution method was used for rotation during factor analysis, finally obtaining the rotated eigenvalue, variance contribution rate, and cumulative variance contribution rate.

**Figure 4:**
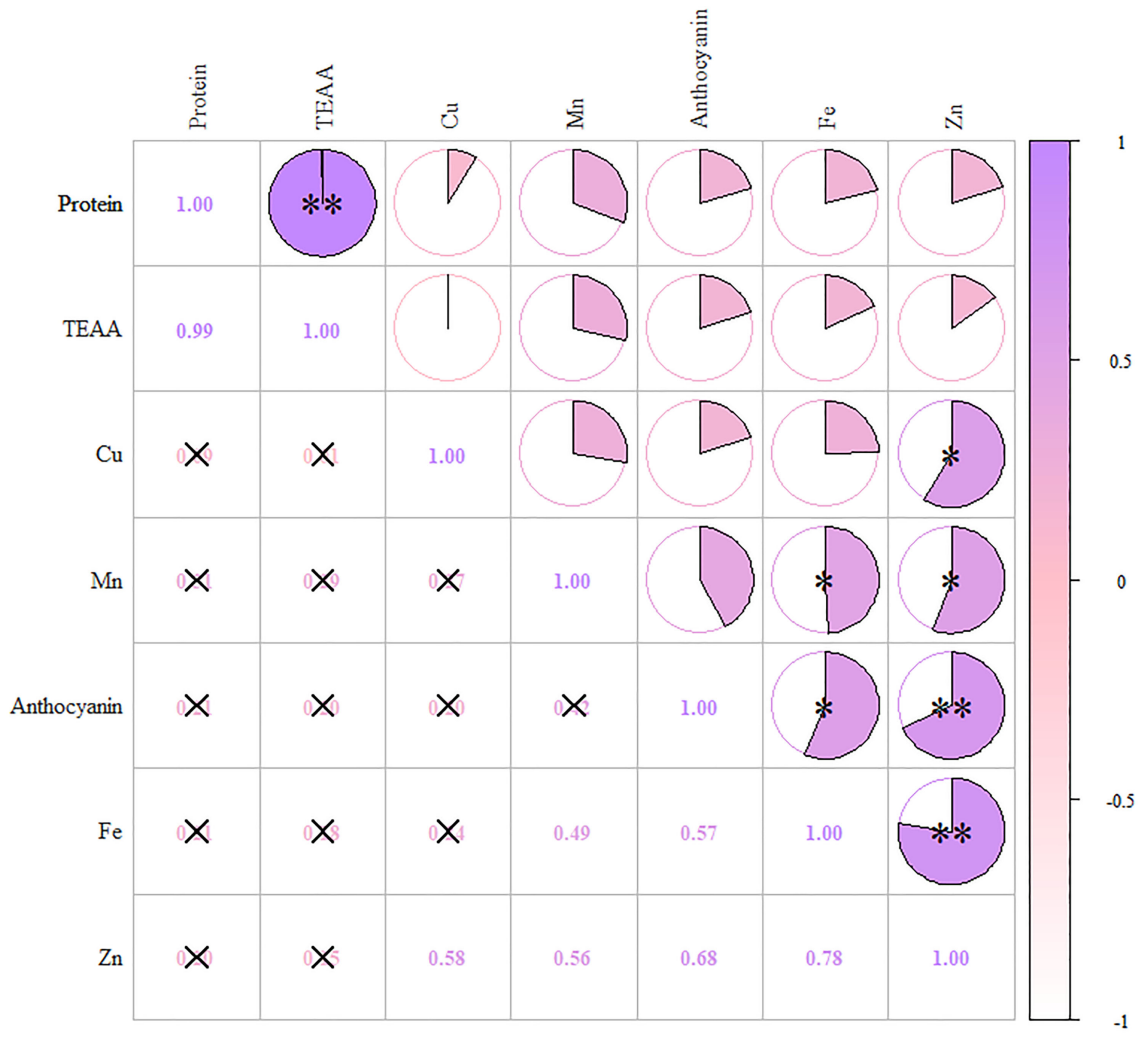
Correlation heatmap of anthocyanins, protein, TEAA, Fe, Zn, Mn and Cu. The color scale from white (low) to purple (high) indicates the correlation coefficient. *means significantly different at p < 0.05, **means significantly different at p < 0.01.

**Figure 5:**
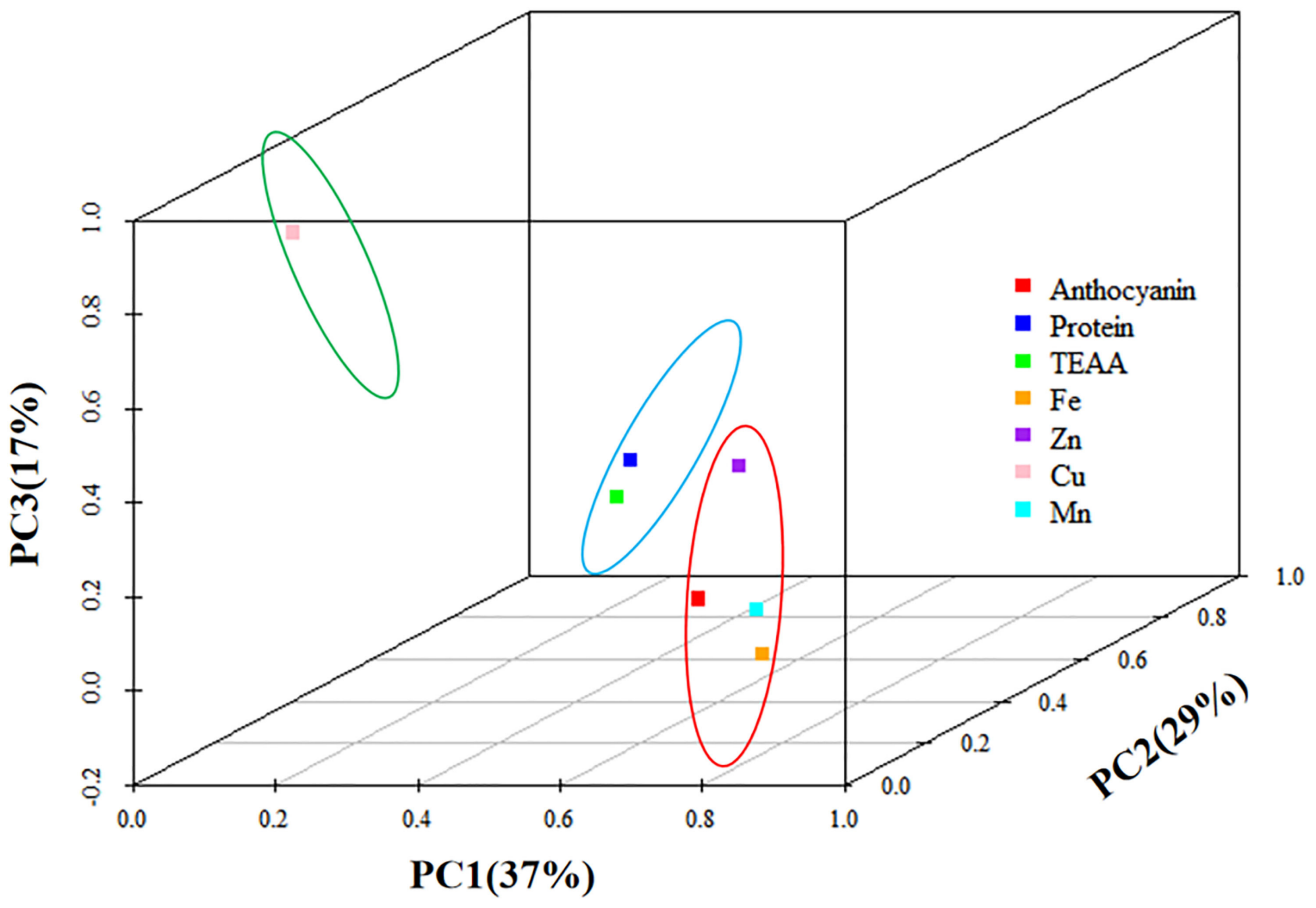
Principal component analysis rotational load diagram of the seven nutrient indices.


In the first principal component, anthocyanins had higher loads, along with Fe, Zn, and Mn, indicating a high correlation (**Figure 4**). The second component mainly contained protein and essential amino acids, while the third component contained only Cu. **Table S4** shows the rotational component matrix of the factor analysis; **Figure 5** presents the rotational load diagram of seven nutrient indices from the PCA. **Table S5** shows the weight score of each index factor in the 18 amphiploids. The scores of the three principal components were calculated by using the standardized data of 7 different nutrient indices, and the comprehensive nutrient scores of the 18 amphiploids were calculated by using the contribution rate of rotation variance as the coefficient. The principal components (Y1, Y2, Y3) and comprehensive score (Y) were calculated according to the formulas below:

$$Y1=0.416\times Z_{Anthocyanin}-0.076\times Z_{Protein}-0.059\times Z_{TEAA}+0.394\times Z_{Fe}+0.267\times Z_{Zn}-0.202\times Z_{Cu}+0.217\times Z_{Mn}$$

$$Y2=-0.067\times Z_{Anthocyanin}+0.504\times Z_{Protein}+0.505\times Z_{TEAA}-0.071\times Z_{Fe}-0.066\times Z_{Zn}+0.009\times Z_{Cu}+0.066\times Z_{Mn}$$

$$Y3=-0.274\times Z_{Anthocyanin}+0.044\times Z_{Protein}-0.034\times Z_{TEAA}-0.167\times Z_{Fe}+0.219\times Z_{Zn}+0.924\times Z_{Cu}+0.034\times Z_{Mn}$$

Y=0.452×Y1+0.345×Y2+0.202×Y3

The comprehensive evaluation scores and rankings resulting from the factor analysis of the seven nutrient indices in the 18 amphiploids served as crucial foundations for assessing their nutritional profile. As shown in **Table S6**, after reviewing the comprehensive scores and rankings, the only yellow-grained amphiploid among the 18 amphiploids ranked last. Among the top five samples, Syn-ABA^b^-5, Syn-ABA^b^-8, and Syn-ABA^b^-11 exhibited elevated scores within the second principal component. This implies heightened levels of protein and essential amino acids, rendering them viable candidates for the cultivation of high-protein and high-amino-acid-content wheat varieties. Conversely, Syn-ABA^b^-4 and Syn-ABA^b^-17 exhibited superior scores within the first principal component. This positioning qualifies them as suitable parents for biofortification breeding, enriching wheat with anthocyanins, Fe, and Zn.

## Discussion

*T. boeoticum* serves as a secondary gene pool for wheat; however, its integration into wheat breeding has been hindered by limited chromosomal pairing between *T. boeoticum* and common wheat (Feldman and Sears, 1981). Generating amphidiploids offers an effective approach to address this problem. Numerous previous studies have aimed to create amphidiploids by crossing wheat with related species from the genera *Aegilops*, *Secale*, *Thinopyrum*, and *Triticum* (Nemeth et al., 2015). These amphidiploids are sought as a source of genetic variation for enhancing wheat (Kroupin et al., 2019). In recent years, fluorescence *in situ* hybridization (FISH) has gained prominence as a crucial technique for cytological identification. In this study, three probes, Oligo-pTa535, Oligo-pSc119.2, and Oligo-pTa713, were used simultaneously to identify A, A^b^ and B genomes, and this was combined with GISH to verify the results. The probes exhibited distinct signals on the chromosomes of the three genomes. These probes can be used as cytological markers in future wheat breeding programs.

Anthocyanins have garnered growing interest among nutritionists and food scientists due to their antioxidant activity. The blue wheat variety Skorpion cultivated in the Czech Republic exhibited a total anthocyanin content of 9.26 mg/kg (Bartl et al., 2015). The anthocyanin content in the whole meal of three Canadian blue wheat species was 135.64, 168.62, and 194.36 μg/g (Francavilla and Joye, 2022). Liu et al. (2022) reported the TAC of the 4A^b^ (4B) disomic substitution line Z18-1244 as 67.24 μg/g. In this study, the TAC of 17 blue-grained amphidiploids was determined to be between 82.830 μg/g and 207.606 μg/g. Variations in anthocyanin content across studies are evident, likely stemming from distinctions in varieties and growth conditions, including soil, weather, and light exposure periods, all of which influence anthocyanin levels (He et al., 2010). The protein content of durum wheat is 12-16%, which is generally higher than that of common wheat (8-14%) (Marcotuli et al., 2020). The protein content of the 18 amphidiploids developed within this investigation ranged from 15.44% to 18.05%. The essential amino acid content plays a pivotal role in food quality assessment (Tian et al., 2018). The SRCs of the amphidiploids were between 57.60 and 64.57, indicating that their amino acid composition was reasonable, and the content of the first limiting amino acid, lysine (3.151-5.027 μg/g), in grains was relatively high. Approximately two billion people worldwide experience micronutrient deficiencies, notably, Fe and Zn deficiencies, and elevating the micronutrients content in grains is an effective solution for this problem (Çakmak et al., 2004). The iron (Fe) and zinc (Zn) concentrations were evaluated for 66 spring and common winter wheat genotypes derived from Central Asian breeding programs. Specifically, the iron content ranged from 25 mg/kg to 56 mg/kg (average 38 mg/kg), while the zinc content ranged between 20 mg/kg and 39 mg/kg (average 28 mg/kg) (Morgounov et al., 2007). The Fe and Zn levels in the blue-grained amphidiploids ranged from 60.55 to 97.41 mg/kg and 39.18 to 57.82 mg/kg, respectively, exceeding the average amount in Asian wheat.

Research has previously indicated that colored wheat tends to accumulate higher levels of micronutrients than white wheat. Tian et al. (2018) pointed out that colored wheat exhibited increased levels of Zn, Fe, and Mg, by 108.54%-142.68%, 8.57%-42.86%, and 5.31%-40.63%, respectively. Guo et al. (2013) observed higher levels of Fe, Zn, Mn, Cu, Se, Mg, K, and P in black wheat. This study obtained results similar to those of previous reports. Correlation analysis and PCA showed that anthocyanins had a high correlation with Fe and Zn, with high loads in the first principal component. This suggests a close association between anthocyanins and Fe and Zn in blue-grained wheat. Since the created amphidiploids came from different parents, we also constructed a nutritional evaluation system based on 7 indices to evaluate, compare and rank these amphiploids. Among the top five amphiploids, Syn-ABA^b^-5, Syn-ABA^b^-8, and Syn-ABA^b^-11 had high protein and amino acid levels, while Syn-ABA^b^-4 and Syn-ABA^b^-17 had high anthocyanin and micronutrients levels. Therefore, the created amphidiploids exhibited the potential to serve as parent candidates for hybridization with common wheat, guided by comprehensive score rankings, toward enhancing the nutritional quality of wheat.

## Data availability statement

The datasets presented in this study can be found in online repositories. The names of the repository and accession number(s) can be found below: PRIDE database (https://www.ebi.ac.uk/pride/), accession number PXD04607.

## Author contributions

XL: Data curation, Investigation, Methodology, Software, Writing – original draft. XJ: Investigation, Software, Methodology, Writing – review & editing. JZ: Investigation, Software, Writing – review & editing. HY: Investigation, Software, Writing – review & editing. MS: Investigation, Software, Writing – review & editing. LW: Investigation, Software, Writing – review & editing. YM: Investigation, Software, Writing – review & editing. LC: Software, Investigation, Writing – review & editing. KZ: Investigation, Software, Writing – review & editing. MH: Software, Supervision, Writing – review & editing. BJ: Investigation, Methodology, Writing – review & editing. LH: Investigation, Supervision, Writing – review & editing. SN: Investigation, Supervision, Writing – review & editing. XJC: Investigation, Data curation, Writing – review & editing. XC: Investigation, Software, Writing – review & editing. DL: Supervision, Methodology, Writing – review & editing. LZ: Conceptualization, Methodology, Project administration, Supervision, Validation, Writing – review & editing.
